# Determination of Coenzyme A (CoASH) in the Presence of Different Thiols by Using Flow-Injection with a UV/Vis Spectrophotometric Detector and Potentiometric Determination of CoASH Using an Iodide ISE

**DOI:** 10.3390/molecules15010100

**Published:** 2009-12-29

**Authors:** Josipa Giljanović, Ante Prkić

**Affiliations:** Department of Analytical Chemistry, Faculty of Chemistry and Chemical Technology, Teslina 10/V, 21000 Split, Croatia

**Keywords:** coenzyme A, flow-injection determination, spectrophotometric determination, potentiometric determination

## Abstract

Coenzyme A (CoA or CoASH) is one of the most important biologically active compounds, and for this reason a reliable, fast and simple determination of this species is needed. We describe a simple and fast assay of CoASH using potentiometric flow-injection analysis and spectrophotometric kinetic determination. The described methods are suitable for use over a wide CoASH concentration range (1×10^-6^ – 1×10^-4^ M).

## 1. Introduction

**Figure 1:**
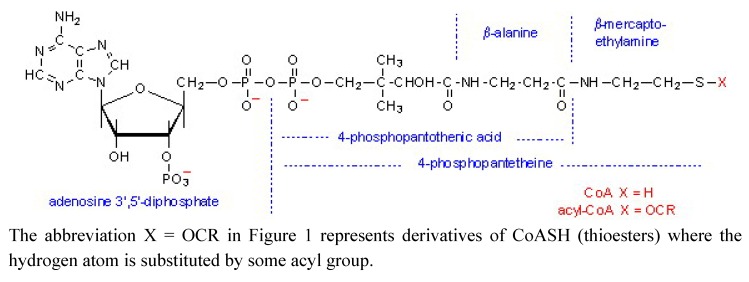
Structure of Coenzyme A.

Coenzyme A (CoASH; Figure 1), is one of the most important biologically active compounds. It is involved in about 100 chemical reactions in cells such as the metabolism of carbohydrates, lipids and amino acids. The most important reaction, which CoASH initiates, is the tricarboxylic acid cycle that produces more than 90% of the energy required for life processes (basal metabolism). CoASH also serves as an acyl carrier in many metabolic reactions due its active thiol (-SH) group which covalently bonds to an acyl group to form thioesters [1].

For these reasons a reliable, fast and simple determination of CoASH is needed. Lots of methods have been described for determination of CoASH, such as high-performance liquid chromatography (HPLC) and high-performance capillary electrophoresis (HPCE) combined with different spectrophotometric detectors, both UV [2] and fluorimetric [3]. HPLC is the most popular and the most widespread technique, but it is also impractical as it generates a lot of different organic wastes and requires a lot of time for the analyses (45–120 min) [4,5]. HPCE is a newly described method with promising results [6,7,8,9,10]. One of the latest methods of CoASH determination is fluorimetric determination based on the reaction of CoASH with a terbium (Tb^3+^) organic complex [11]. There are also methods which include radioisotope atoms in the structure of some derivatives of CoASH [12,13].

In the literature many methods of determination of different thiols using flow-injection with a UV/visible spectrophotometric detector have been described [14,15]. Because of the thiol group in its structure, CoASH can also be considered a thiol compound and there are several articles describing determination of CoASH derivatives using flow-injection with UV/visible spectrophotometric detection [2,16]. In our laboratory we have already carried out determinations of a few thiols (*etc*. *N*-acetyl-L-cysteine, DL-pencillamine, L-cysteine, L-glutathione) using flow-injection coupled with a potentiometric detector [17,18,19,20]. Herein we describe the determination of CoASH using a UV spectrophotometric detector at 258 and 510 nm and flow-injection with a potentiometric detector.

## 2. Results and Discussion

### 2.1. Flow-injection determination of CoASH using a potentiometric detector

The characteristics of the flow-injection analysis (FIA) system were optimized: carrier-stream (0.1 M HClO_4_) of 2.7 mL·min^-1^ flow-rate, injection frequency of 3 min, injection time 30 s and the reagent solution used to ensure a constant pH value and ionic strength was silver nitrate, *c*(AgNO_3_) = 5×10^-5^ M in 0.1 M HClO_4_. Tested solutions of CoASH were injected into the carrier stream with a 200 μL loop volume loop injection valve. The potentiometric response of the cell to the analyzed compounds was monitored with a mV-meter (recorder) and potentiometric data was captured continuously with a personal computer. For the experimental measurements a two-channel FIA setup has been used. For determination of CoASH a potentiometric sensor based on the tubular electrode [22,23] with an internal diameter of 2.0 mm and a length of sensing area of 1.2 mm was used.

### 2.2. Flow-injection system optimization

The tubular electrode and reference electrode are located downstream after mixing of two channels. A constant representing a dilution of the sample or reagent after mixing of two solutions depends on the flow rates in channels and can be analyzed and expressed as follows:

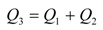
(1)

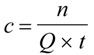
(2)

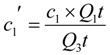
(3)

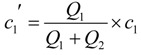
(4)
*Q_1_, Q_2_, Q_3_* - flow-rates (mL min^-1^); *c* – concentration of sample (mol L^-1^); *c*_1_ – concentration of sample after injection in the flow system (mol L^-1^); *c*_1_’ – concentration of sample after injection and mixing in the flow system (mol L^-1^)

**Figure 2:**
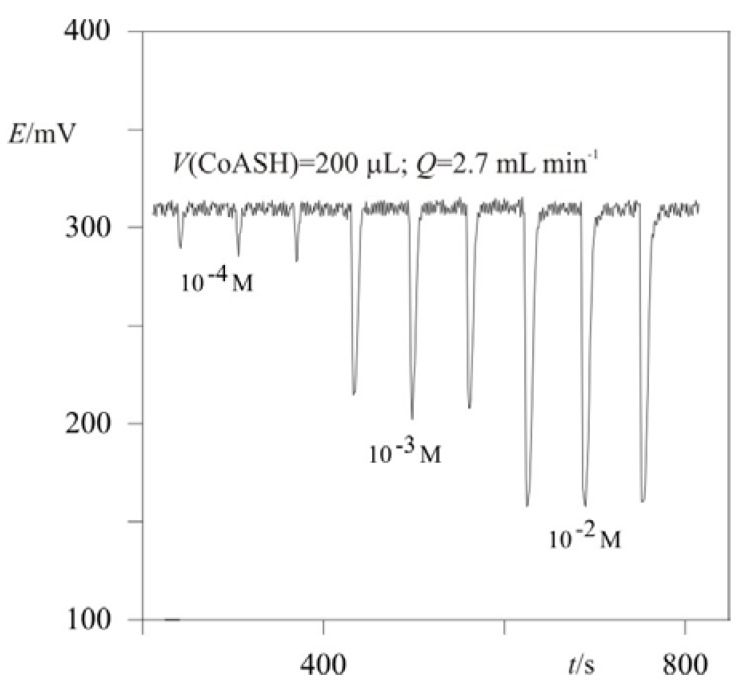
Recording of peaks obtained from CoASH injection into a carrier stream.

In our experiment, the same flow-rates, *Q_1_* = *Q_2_* = 2.7 mL·min^-1^, were used, and the reagent or sample dilution was 0.5 [equation (4)]. Figure 2 shows a typical CoASH response in the FIA system for a concentration range from 10^-4^ to 10^-2^ M. All concentrations were injected in triplicate. The obtained peaks are sharp and reproducible, displaying a fast return to baseline, which is stable.

**Figure 3:**
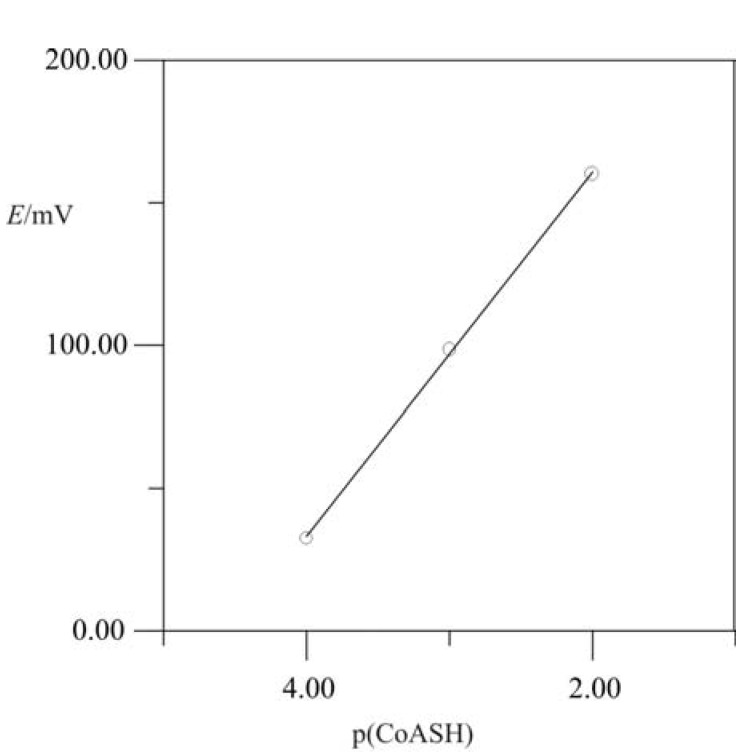
Response of CoASH in flow-injection system with iodide ion-selective electrode (ISE) as detector. Experimental values are represented with dots and calculated ones with a straight line.

When the recorded signal heights (in mV) are plotted against the negative logarithms of CoASH in the injected sample (Figure 3), we obtain a value of 63 mV, which is acceptable according to the theoretical value for monovalent ions (59 mV). RSD for the tested concentration range was 0.9995.

### 2.3. “Classic” (Batch experiment) potentiometric determination of CoASH

The response of the home-made iodide ion-selective electrode to Ag^+^ was monitored by the method of continuous dilution of silver ions in the reaction vessel. Serial dilution 1.0×10^-1^ mol·L^-1^ of the standard silver nitrate solution was performed using 0.1 M perchloric acid up to *c*(Ag^+^) = 6.3×10^-6^ mol· L^-1^, (pAg = 5.2). Standard CoASH solution was added in the same perchloric acid solution and the response of the electrochemical cell to CoASH was tested by the method of continuous decrease of CoASH. During measurement, the solution was constantly mixed and temperature was kept constant at 25 °C and 40 °C.

**Figure 4:**
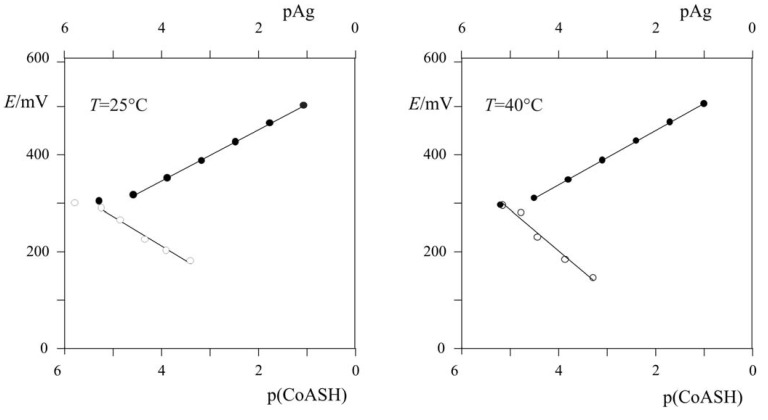
CoASH determination using classic potentiometric with constant mixing at (a) 25 °C and (b) 40 °C ● - Ag, ○ - CoASH.

Points on the graph (Figure 4) represent experimental data and straight lines have been calculated by using the linear regression method. It can be seen that the home-made iodide electrode follows the changes of Ag^+^ and CoASH concentration linearly over a wide concentration range. A stable potential was reached in about 1 minute. At 25 °C a potential change of 53 mV per decade concentration change of Ag^+^ was recorded with a RSD of 0.9788 and a potential change of 60 mV per decade concentration change of CoASH was recorded with a RSD of 0.9878. At 40 °C a potential change of 56 mV per decade concentration change of Ag^+^ was recorded with a RSD of 0.9899 and a potential change of 84 mV per decade concentration change of CoASH was recorded with a RSD of 0.9799. The home-made iodide ion-selective solid-state electrode had a limit of detection of CoASH of 6.3 × 10^-6^ mol·L^-1^ with linear response range of *c*(CoASH) ≅ 10^-5^ –10^-3^ mol·L^-1^.

Limit of detection (LOD) was calculated (both potentiometric and spectrophotometric measurements) by using the following formula:

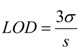
(5)
*σ*- standard deviation; *s* – slope of curve

#### 2.3.1. Calculating *K_sp_*(CoAS-Ag)

Linear Response Range

In our experiments, the “home-made" iodide ISE responds primarily to the activity of the silver ion at the sample solution-electrode membrane interface downstream after mixing of two channels. The potential of the cell with the sensing electrode is given by:

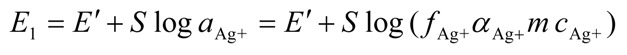
(6)
where *S*, *f*, *α*, *m* and *c* denote the response slope of the electrode, the activity coefficient, the fraction of Ag^+^, the dilution constant and the total or analytical concentration of silver ions in reagent solution respectively.

In the absence of ions in the streaming solution that form sparingly soluble silver salts or stable silver complexes and at constant ionic strength the potential of the sensor can be expressed by the following equation:

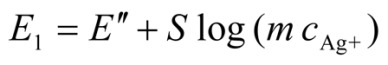
(7)

When a sample containing CoASH at a sufficiently high concentration to cause precipitation of CoASAg is injected into the carrier stream, the silver ion concentration will be lowered to a new value. If *d m c*(CoASH) >> *m c*_Ag+_, where dispersion of the sample is represented by the constant *d*, the free silver ion concentration at equilibrium can be analyzed and expressed as follows:

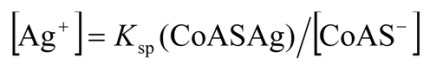
(8)

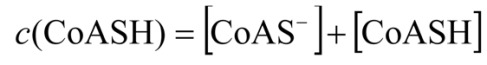
(9)

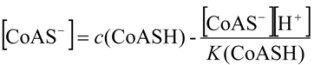
(10)

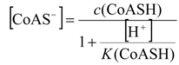
(11)

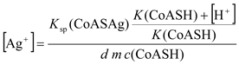
(12)
where *K*_sp_ is the solubility product of silver salt while *K* is the dissociation constant of sulfhydryl group, *K*=[CoAS^-^][H^+^]/[CoASH].

In the flow-injection measurement of compounds with highly reactive sulfhydryl group, the potential of the peak may be described by following equation:

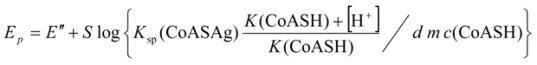
(13)

Since the peak height, *h*, in these measurements is equal to the potential difference:

*h* = *E*_1_ - *E*_p_ (14)

using equation (7) one can obtain the equation for peak height. Hence, *f*, *d*, *m*, [H^+^] and *c*_Ag+_ are kept constant and *d m c*(CoASH) >> *m c*_Ag+_, a linear dependence between the peak height and logarithm of concentration of CoASH with the slope of 59 mV {p(RSH)}^-1^ can be obtained.

When a sample contains RSH forms of Ag(SR)_n_^(1-n)+^ complexes instead of precipitates, the equilibrium concentration of Ag^+^ ions is also lowered. Hence, if injected concentration of RSH is much higher than silver concentration in streaming solution the potential of the peak may be described by the following equation:

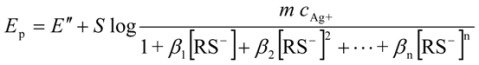
(15)
where *β* the stability constant and [RS^-^] is the free concentration of ligand. The concentration of ligand can be expressed by:

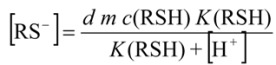
(16)

If *d*, *m*, *c*_Ag+_ and [H^+^] are kept constant and *d m c*(CoASH) >> *m c*_Ag+_, a linear dependence between the peak height and logarithm of *c*(CoASH) may be obtained, but only if in the denominator of equation (15) one term predominates and the "1" can be neglected. The slope of the potentiometric response will be *n S* mV {p(CoASH)}^-1^ where *n* is the number of ligands in the predominant complex.

The response of the electrode with the membrane consisting of pure silver halides AgX or AgX mixed with Ag_2_S to anions which form sparingly soluble salts or stable complexes with Ag^+^ has been discussed by Morf *et al*. [24]. In our experiment the potential response of the utilized potentiometric detector to CoASH was in agreement with the theoretical value for monovalent electrode.

#### 2.3.2. Determination of Solubility Product of CoASAg or Stability Constant of Ag(SAoC)n^(1-n)+^

If we apply continuous-flow instead of a flow-injection technique the dispersion of the sample can be neglected and the potential of the peak would reach a stable value. When the streaming solution contains insoluble CoASAg and CoASH in excess, the next equilibria exist in solution: 
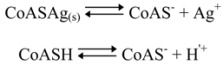
 and the solubility product of CoASAg, when pH is fixed and known, can be calculated using equation (17):

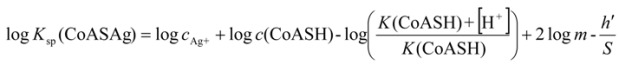
(17)

The solubility product of CoASAg has been calculated by using equation (17) in a continuous-flow experiment at pH = 1 (Table 1). It can be seen that ***K*_sp_** gathered by potentiometric determination by using laboratory home-made electrode and commercial purchased iodide ISE are very similar but slightly different. It can be explained with fact that membrane of our laboratory home-made electrode and commercial purchased iodide ISE differ in morphology and the chemical composition of the membrane [22,23].

**Table 1:** Comparation of p*K*_sp_.

Used electrode	p*K*_sp_	*K*_sp_
Laboratory home-made electrode with silver pellet	18.4	4.0×10^-19^
Commercial iodide ISE	18.7	2.0×10^-19^

### 2.4. Spectrophotometric determination of CoASH

**Figure 5:**
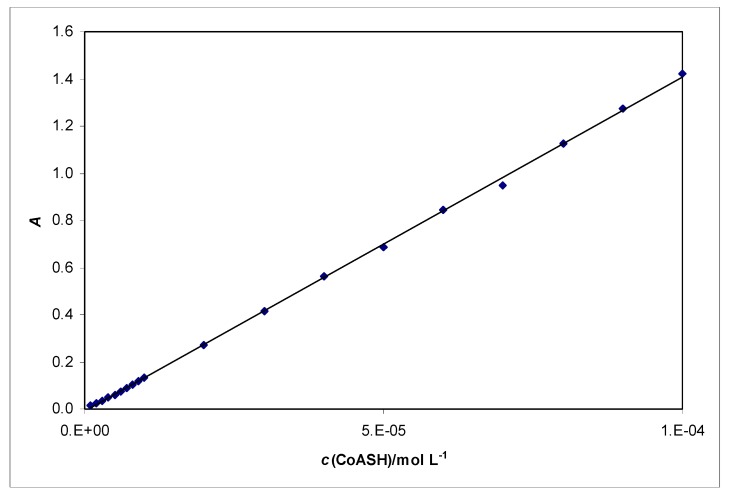
Linear response range of CoASH in concentration range 1.0x10^-6^ to 1.0x10^-4^ M.

In the literature different spectrophotometric methods are described for determination of the concentration of CoASH [2,11]. Here we have described a novel method for determination of CoASH using a UV/Vis spectrophotometer. We have found out that CoASH absorbs at 258 nm. The linear response range for CoASH at 258 nm is from 1.0 × 10^-6^ to 1.0 × 10^-4^ M, and limit of detection is 7.3 × 10^-7^ M (Figure 5.) Molar absorptivity of CoASH is 14 328 ± 167 L·mol^-1^ cm^-1^, *n* = 5.

We analyzed the absorbance of CoASH over a wide pH range. CoASH has an acceptable constant absorbance at 258 nm through a wide pH range (Table 2). A reasonable explanation of this phenomenon can be found in the structure and complexity of the CoASH molecule.

**Table 2:** Absorbance of CoASH at 258 nm, *c*=10^-4^ M in wide pH range.

**pH**	1.00	2.00	3.00	4.00	5.00	6.00	7.00	8.00	9.00	10.00	11.00	12.00	13.00
*A*	1.42	1.41	1.41	1.39	1.38	1.38	1.40	1.40	1.39	1.39	1.43	1.43	1.42

We analyzed the absorbance of the following thiols: *N*-acetyl-L-cysteine (NAC), glutathione (glu), penicillamine (pen), cysteine (cys) over the concentration range from 1.0 x 10^-5^ to 1.0 x 10^-4^ M and at a wavelength of 258 nm (Table 3). In Table 3 it can be seen that absorbance of analyzed thiols have acceptable zero values and can be neglected.

**Table 3:** Absorbance of different thiols at 258 nm.

Thiols	*A*_258_		
	*c*=10^-5^ M	*c*=8.0×10^-5^ M	*c*=10^-4^ M
CoASH	0.143	1.142	1.429
cys	-0.005	0.001	0.002
glu	-0.003	0.005	0.008
NAC	0.003	0.008	0.008
pen	-0.006	-0.002	-0.001

**Figure 6:**
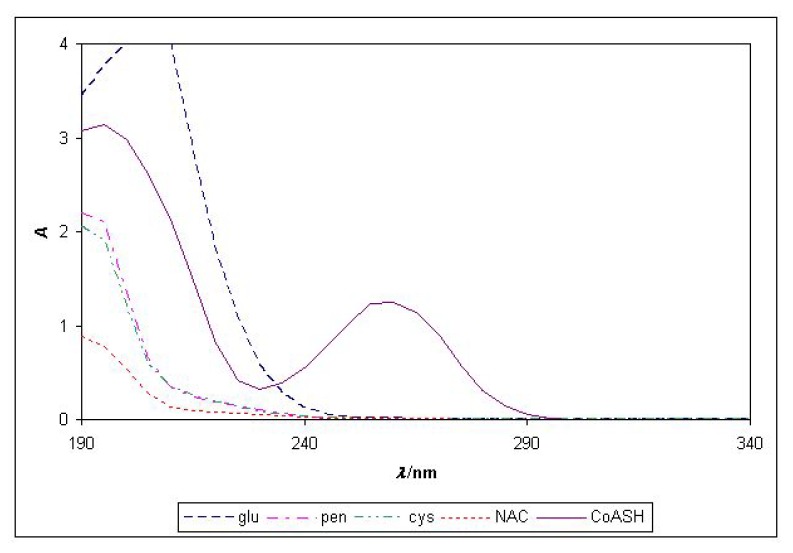
Absorption spectra of different thiols from 190 to 340 nm, *c* = 1.0x10^-4^ M, *λ*_max_ (CoASH) = 258 nm.

It can be seen that only CoASH absorbs at 258 nm and we recommend this wavelength for determination of CoASH in mixtures with the analyzed thiols (Figure 6).

### 2.5. Kinetic determination of CoASH with a UV/Vis spectrophotometer as detector

**Figure 7:**
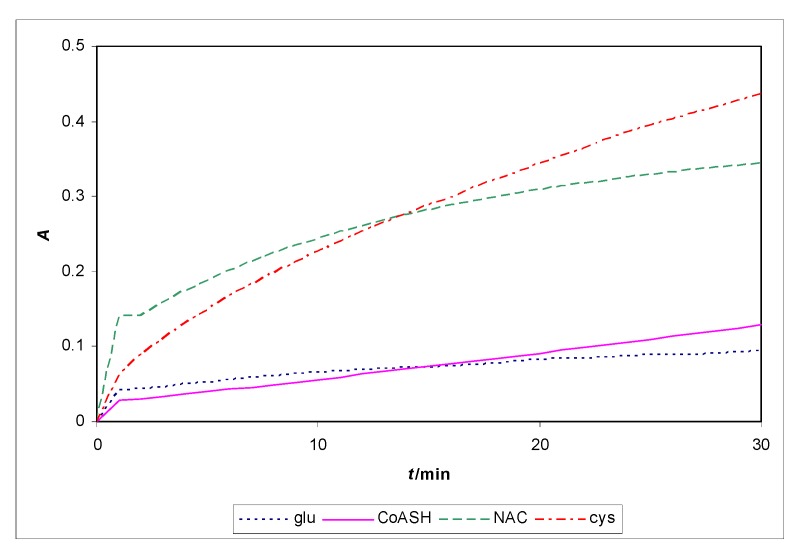
Absorbance change during reaction of thiols with Fe^3+^ at 510 nm and pH=2.8.

Kinetic spectrophotometric determination of different thiols in their reaction with Fe^3+^ and 1,10-phenothroline at 510 nm at pH = 1 and pH = 2.8 has been used in a flow system. The system has been optimized at a flow-rate of 7 mL·min^-1^ and the thiol compounds were injected 60 seconds after the experiment had started. Reaction volume was 50 mL, mixed with a magnetic agitator. Concentrations of thiol compounds were 8.0 × 10^-5^ M, concentration of Fe^3+^ was 8.0 × 10^-4^ M and concentration of 1,10-phenanthroline was 3.2 × 10^-3^ M. Kinetic measurements last for 30 min. Tested thiols were: glutathione, penicillamine, cysteine, NAC and CoASH. At pH = 1, all tested thiols show low reduction power probably because of their protonated thiol groups and that is reason why the absorbance does not change more than 0.05 absorbance units for all tested thiols. At pH = 2.8 the tested thiols show different reduction powers: CoASH and glutathione show low reduction power and *N*-acetyl-L-cysteine and cysteine show moderate reduction power, Figure 7.

## 3. Experimental

### 3.1. Chemicals

All required solutions were prepared by dissolving a certain amount of solid chemical in Suprapure water. Suprapure water (declared conductivity 0.04 μS cm^-1^) was prepared by a Millipore Simplicity (USA) unit. Potassium iodide, KI, p.a., iron(III)-chloride hexahydrate, FeCl_3_x6H_2_O, p.a., silver nitrate, AgNO_3_, p.a. and 1,10-phenantroline monohydrate, C_12_H_8_N_2_xH_2_O, p.a. were obtained from Kemika (Croatia). Perchloric acid, HClO_4_, p.a., L-glutathione, C_10_H_17_N_3_O_6_S, p.a. and L-cysteine, C_3_H_7_NO_2_S, p.a. were obtained from Merck (Germany). *N*-acetyl-L-cysteine, C_5_H_9_NO_3_S, p.a. and DL-penicillamine, C_5_H_11_NO_2_S, p.a. were obtained from Fluka (Germany). Coenzyme A trihydrate, C_21_H_36_N_7_O_16_P_3_Sx 3H_2_O, p.a. was obtained from Sigma (Germany).

### 3.2. Preparation of solutions

Coenzyme A solution was prepared by dissolving the required amount of CoASH in prechloric acid, *c*(HClO_4_)=0.100 mol·L^-1^. Concentration of CoASH base solution was *c*(CoASH)=1.0 × 10^-3^ mol·L^-1^.

Perchloric acid solution, *c*(HClO_4_)=0.100 mol·L^-1^ was prepared from concentrated perchloric acid (*w* = 60%, *ρ* = 1.53 kg·L^-1^).

Silver nitrate solution was prepared by dissolving the needed amount of AgNO_3_ in perchloric acid, *c*(HClO_4_)=0.100 mol·L^-1^. Concentration of AgNO_3_ base solution was *c*(AgNO_3_)=1.0 × 10^-1^ mol·L^-1^.

Potassium iodide solution was prepared by dissolving the appropriate amount of KI in perchloric acid, *c*(HClO_4_)=0.100 mol·L^-1^. Concentration of KI base solution was *c*(KI)=1.0 × 10^-1^ mol·L^-1^.

Iron(III) chloride solution was prepared by dissolving the needed amount of FeCl_3_x6H_2_O in perchloric acid, *c*(HClO_4_)=0.100 mol·L^-1^. Concentration of FeCl_3_ base solution was *c*(FeCl_3_)=1.0 × 10^-2^ mol·L^-1^.

1,10-Phenantroline solution was prepared by dissolving a suitable amount of 1,10-phenantroline monohydrate in perchloric acid, *c*(HClO_4_)=0.100 mol·L^-1^. Concentration of 1,10-phenantroline base solution was *c*(1,10-phenantroline)=1.0 × 10^-2^ mol·L^-1^.

Thiol solutions were prepared by dissolving the needed amount of a thiol (*N*-acetyl-L-cysteine, L-glutathione, DL-penicillamine and L-cysteine) compound in perchloric acid, *c*(HClO_4_)=0.100 mol·L^-1^. Concentration of a thiol compound base solution was *c*(thiol compound)=1.0 × 10^-2^ mol·L^-1^.

### 3.3. Apparatus

The basic design of the tubular flow-through electrode unit was the same as previously described [22]. All parts of the multi-purpose solid-state electrode body were made from PTFE or stainless steel. A pressed pellet with a hole drilled in the center (2.0 mm i.d. and 1.2 mm long) was incorporated in the electrode body. The preparation and performance of the silver-iodide-based pellet hydrophobized by PTFE have been described previously [23]. A coaxial cable and one stainless-steel part of the electrode body provided electrical connection between the pellet and milivoltmeter. In all potentiometric measurements as reference electrode was used an Orion 90-02 double junction reference electrode with characteristics of saturated calomel electrode.

**Figure 8:**
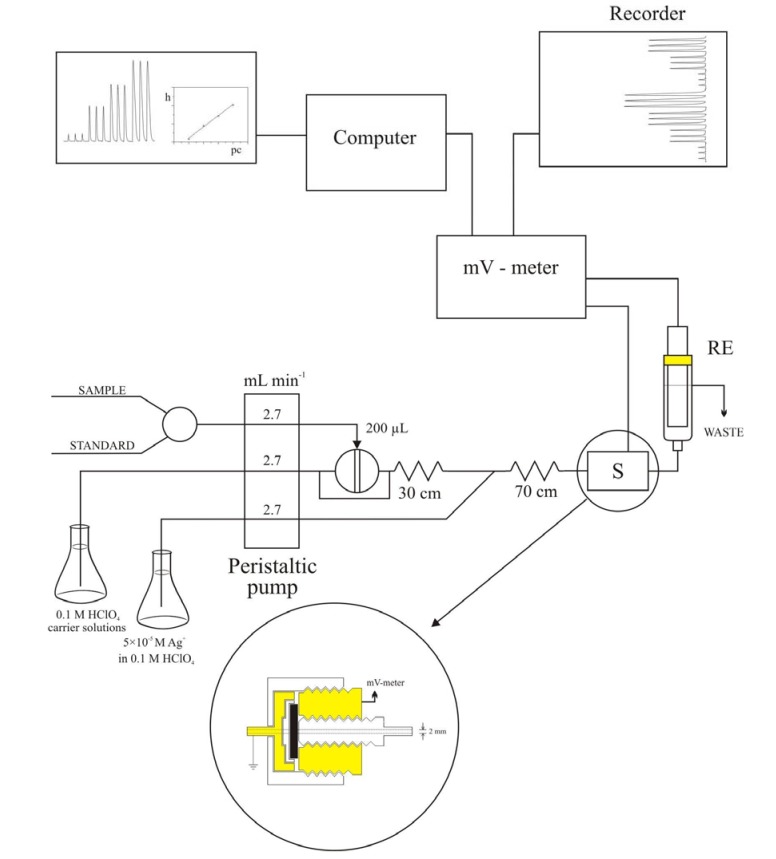
Schematic image of flow-injection system with tubular potentiometric sensor based on AgI.

The tubular indicator electrode has been used with an Orion 90-02 double junction reference electrode, placed in a vessel downstream from the indicator electrode just before the solution has gone to waste. Potentiometric data has been recorded at room temperature with a milivoltmeter (Model MA 5740, Iskra, Ljubljana, Slovenia) coupled to a personal computer and recorder. The tubular flow-through electrode has been incorporated into setup of the flow injection system as shown in Figure 8.

**Figure 9:**
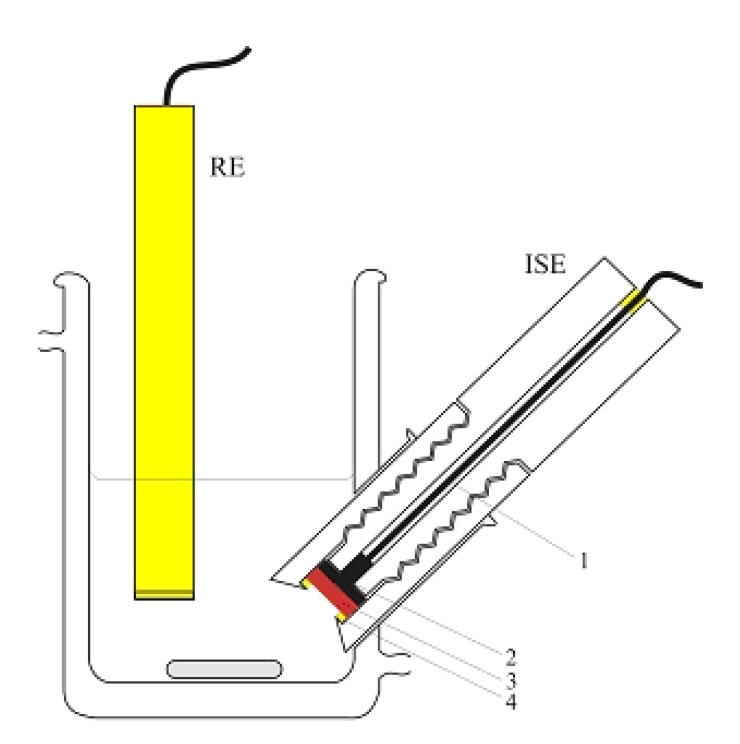
Schematic image of “batch” experiment. 1) coaxial cord 2) solid-state disk made of stainless steel 3) AgI based sensor made by chemical treatment Ag plate of pure silver 4) silicon rubber**.**

The flow system consisted of a peristaltic pump (type HPB 5400, Iskra, Ljubljana, Slovenia) fitted with silicone rubber tubing of 2.0 mm i.d. and an injection valve (model V-100, Tecator, Sweden). The outlet of the injector has been connected to the flow cell via silicone tubing of 0.5 mm i.d. The detector for spectrophotometric measurements was a Shimadzu UV-1601 spectrophotometer. Figure 8 shows a schematic of the FIA system, RE – reference electrode (Orion 90-02 DJRE), S – sensor (home-made iodide ion-selective electrode). Figure 9 is a schematic of a “batch” experiment.

**Figure 10:**
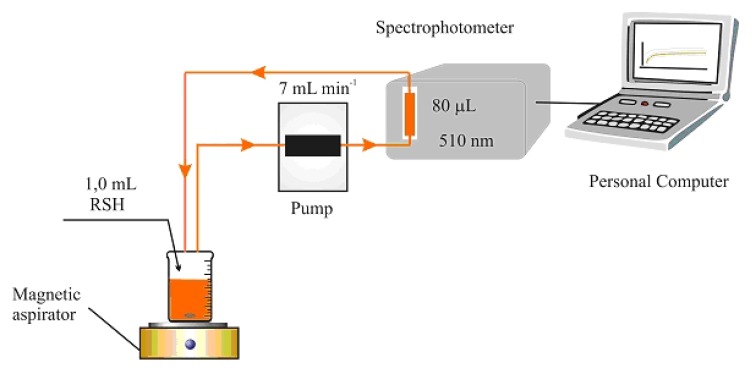
Flow system with a UV/Vis spectrophotometer as detector.

Figure 10 is a schematic of the kinetic determination experiment with flow direction.

## 4. Conclusions

A home-made iodide ion-selective electrode is acceptable as detector in a flow-injection system for determination of CoASH in perchloric acid, pH = 1, over the concentration range *c*(CoASH) = 10^-4^–10^‑2^ mol·L^-1^ with a potential change of 63 mV per decade change of concentration of CoASH. Under the experimental conditions CoASH forms a very insoluble compound in reaction with Ag^+^ from the surface of membrane or from the solution mass. From experimental data (Table 1) *K*_sp_ was calculated by assuming that CoASH and Ag^+^ form a compound with 1:1 stoichometry. Measurement data obtained from batch experiments with home-made iodide ion-selective electrode are in very good agreement with ones obtained from flow-injection analysis. The slope of the linear part of curve was 60 mV per decade change of concentration of CoASH over the concentration range *c*(CoASH) = 2 × 10^-6^-4.7 × 10^-4^ mol·L^-1^. A temperature effect was also observed in “classic” potentiometric experiments with measurements at 25 °C and 40 °C. The slope of experimental curves was 60 mV and 84 mV per decade change of concentration of CoASH, respectively. This can be explained by the fact that in the reaction CoASH and Ag^+^ form a stable complex with a stoichiometry of 1:1.4 at 40 °C.

Spectrophotometric determination at wavelength of 258 nm is very suitable for determination of CoASH over the concentration range *c*(CoASH) = 10^-6^–10^-4^ mol·L^-1^ and the limit of detection is 7.3 × 10^-7^ mol·L^-1^. Molar absorptivity for CoASH was calculated as 14 328 ± 167 L·mol^-1^·cm^-1^, *n* = 5 and it is acceptably constant through a wide range of pH values. Furthermore, at a wavelength of 258 nm we are able to determine CoASH in a mixture of different thiols because at that wavelength the other tested thiols (NAC, penicillamine, cysteine, glutathione) do not interfere up to concentrations of 10^-4^ mol·L^-1^. In flow-injection measurements with spectrophotometer as detector, the reduction ability of CoASH was tested in the reaction with Fe^3+^ at pH = 1 and pH = 2.8 at 510 nm and CoASH showed poor reduction ability.
